# Betting Against the Odds: The Mysterious Case of the Clinical Override in Risk Assessment of Adult Convicted Offenders

**DOI:** 10.1177/0306624X211049181

**Published:** 2021-10-06

**Authors:** Julien Frechette, Patrick Lussier

**Affiliations:** 1 Université Laval, Quebec City, QC, Canada; 2 Centre International de Criminologie Comparée, Montreal, QC, Canada

**Keywords:** clinical override, risk assessment, corrections, decision tree algorithms, level of service and case management inventory, violent offenders

## Abstract

Various tools were designed to guide practitioners in the risk assessment of offenders, including the Level of Service and Case Management Inventory (LS/CMI). This instrument is based on risk assessment principles prioritizing the actuarial approach to clinical judgment. However, the tool’s architects allowed subjective judgment from the practitioners—referred to as clinical override—to modify an offender’s risk category under certain circumstances. Few studies, however, have examined these circumstances. Therefore, the current study used decision tree analyses among a quasi-population of Quebec offenders (*n* = 15,744) to identify whether there are offenders more likely to be subjected to this discretion based on their characteristics. The results suggest that, although the override is rare, it occurred under few specific combinations of circumstances. More precisely, these findings propose that the utilization of the clinical override stems from a perceived discrepancy between risk prediction and management.

In Canada, individuals sentenced to imprisonment or probation are systematically subjected to a risk-and-needs assessment that fulfills several classification and prediction objectives. One of the key objectives is to gather systematic information that will inform the criminal justice practitioner (CJP) responsible for developing and proposing intervention programs tailored to the offender’s risk and needs. In Canada, these risk-and-needs assessments are generally aligned with the principles and guidelines set by the Risk-Needs-Responsivity model (RNR; Andrews et al., 1990). This model was specifically designed for corrections to guide the level of service offered to offenders. To support CJPs, tools based on the RNR model and principles have been developed, such as the Level of Service and Case Management Inventory (LS/CMI; Andrews et al., 2004). This instrument is based on risk assessment principles prioritizing the actuarial assessment approach. Despite the instrument’s strong actuarial component, it also allows, to some extent, the CJP’s subjective judgment. This discretion, known as the clinical override, grants CJPs the possibility to reconsider an offender’s actuarial risk category in an upward or downward fashion. This form of discretionary power—focus of the current investigation—is, therefore, a compromise between actuarial and clinical approaches to risk.

## Literature Review

The clinical override is an important but largely understudied discretionary power attributed to CJPs working in corrections. This feature is the product of several years of research and debates about the relative superiority between actuarial-based (also, mechanical, statistical) risk assessment and those based on human judgment (also, clinical, unstructured clinical). While the results of meta-analyses comparing actuarial and clinical approaches appeared to have dismissed the latter because of its low predictive validity (e.g., Hanson & Morton-Bourgon, 2009; Meehl, 1954), recent studies have somewhat revived the debate. Indeed, despite its relative predictive superiority, several years of research on actuarial risk assessment have highlighted several shortcomings related to prediction and treatment (e.g., Hart et al., 2007; Lussier et al., 2011). The limitations of early actuarial assessments led to various innovations, such as the inclusion of dynamic, potentially changeable risk factors (e.g., Gendreau et al., 1996) as well as the inclusion of criminogenic dynamic risk factors that can be targeted by treatment programs and interventions (e.g., Andrews et al., 2006). These innovations were not enough for some researchers who proposed a more contrasting alternative to the actuarial approach that was framed on structured clinical judgment (also, professional judgment; e.g., Webster et al., 1997).

Proponents of the actuarial approach who also recognized the importance of clinical judgment in risk assessment have proposed another alternative to common risk assessment principles. Acknowledging the limitations of a strict actuarial and clinically unstructured method, Andrews et al. (1990) proposed a somewhat middle-ground approach by adding a clinical override component to an existing actuarial instrument, the Level of Service Inventory-Revised (LSI-R; Andrews & Bonta, 1995). The LSI-R eventually paved the way for the LS/CMI (Andrews et al., 2004), a fourth-generation risk assessment tool endorsing the most recent advancements in research (at the time of its development). Although the LS/CMI is an upgraded and modified version of the LSI-R, the former still allows the use of the clinical override feature. This feature allows CJPs to adjust an offender’s risk category based on considerations deemed important that are not adequately measured or considered at all by the actuarial tool.

The use of the clinical override remains somewhat controversial because it leaves room for unstructured clinical judgment, which has been repetitively proven to not adequately predict recidivism. It has been argued that, while the clinical override is a well-intended feature, it is a fundamentally flawed discretion. For example, Girard (1999) studied the Level of Service Inventory-Ontario Revised (LSI-OR; Andrews et al., 1995), a newly modified risk assessment tool at the time of the publication. Despite the small sample of offenders subjected to a clinical override (OSCO; *n* = 19; 2.7%), the author observed an improvement in the predictive validity of the tool with the override. However, using the same instrument with a larger sample (*n* = 38,689), Orton (2014) showed that the clinical override (AUC = 0.456) had poorer predictive capacities than the actuarial component of the LSI-OR (AUC = 0.740; for similar conclusions, see Duwe & Rocque, 2018; Guay & Parent, 2018; Hogg, 2011; Krauss, 2004).^1^ Nonetheless, several jurisdictions (e.g., the province of Ontario and until recently, the province of Quebec) use a risk assessment tool authorizing the clinical override. In other words, Andrews et al.’s (1990) suggestion that the CJP’s override should not only be a fundamental principle of risk assessment, but also a supplement to risk and need factors when special situations arise seems commonly accepted, especially in Canada.

To our knowledge, the use of the clinical override in risk assessment contexts has been the subject of slightly more than 20 empirical studies, but the scope of these investigations has been limited. From one study to the next, override rates varied considerably. The estimated rates of OSCO among adult generic offenders in clinical and correctional settings oscillated between 6.5% and 16.5% (e.g., Guay & Parent, 2018; Wormith et al., 2012). The use of the clinical override with young offenders, however, is less consistent with rates varying between 7.0% and 58.7% across studies (e.g., McCafferty, 2017; Schmidt et al., 2016; Vaswani & Merone, 2014). These studies also highlighted the fact that the clinical override is typically used to increase an offender’s criminal recidivism risk beyond what is suggested by a risk assessment tool (e.g., Brews, 2009; Storey et al., 2012). Others, nevertheless, have observed more downward risk modifications (e.g., Gore, 2007). Of importance, the clinical override has been examined with a plethora of instruments making it difficult to draw firm conclusions regarding its use beyond the peculiarities of the tools, especially its target population (e.g., youths, adults, sex offenders, probationers, etc.).

A limited number of empirical studies have been conducted to examine the circumstances in which CJPs use the clinical override feature of an actuarial risk assessment instrument. In addition, these studies have been directed mainly using bivariate statistical analyses, therefore, not allowing the identification of combination(s) of circumstances in which individuals are more likely to be subjected to a clinical override (e.g., Guay & Parent, 2018; Wormith et al., 2012). For example, Orton et al. (2021) noted that, in their sample, about half (49.8%) of convicted sex offenders were subjected to a clinical override and almost all of those (98.8%) were used to increase the offender’s risk category. In fact, across different studies, several researchers made similar observations about the relatively high likelihood of convicted sex offenders to be subjected to a clinical override^2^ (e.g., Carns & Martin, 2011; Cohen et al., 2016). Regarding the offender’s disposition, Brews’s (2009) study on a cohort of female offenders under the jurisdiction of Ontario’s correctional services (*n* = 2,831) revealed an over-representation of OSCO among offenders serving a sentence in the community. Studies have also found that males are more likely to be subjected to a clinical override than females (e.g., Chappell et al., 2013; Cohen et al., 2016), especially in an upward fashion (see Orton et al., 2021). Another factor that has been associated with the use of the clinical override relates to the actuarial risk assessment score. By examining the effect of the clinical override on the tool’s predictive validity among a sample of 1,770 sex offenders, Gore (2007) noticed that clinical overrides tended to occur when the actuarial score is at the limit of the cut-off of the previous or subsequent risk category (e.g., low, moderate, high). For example, using a fictional risk assessment instrument, an offender with an actuarial score of 5 (moderate-risk) would be more likely to experience an override (downward), knowing that the low-risk category ranges from scores 1 to 4. Hence, this offender’s actuarial score is only one point away from the previous risk category. The same trend was also noticed in the Guay and Parent’s (2018) study. However, according to them, this tendency was specific to downward overrides.

While these findings showed that the nature and magnitude of an offender’s risk may impact the presence of an override, they also suggested that this tendency may be influenced by other individual characteristics and circumstantial factors. This makes even more sense when one considers the conclusions of Wormith et al. (2012) and Orton (2014) highlighting the presence of a discrepancy between the factors associated with the decision to rely on the clinical override feature of an instrument and those factors predictive of criminal recidivism. In other words, CJPs tended to grant more relevance to certain factors that are not necessarily empirically related to criminal recidivism, therefore, raising concerns about the factors that may influence CJPs’ decision to modify an offender’s risk category.

## Study Aim

While it is a relatively powerful discretionary power, the clinical override also is a key feature of the LS/CMI that can significantly impact an offender’s trajectory within the criminal justice system (e.g., risk category, intensity of supervision and intervention, and parole). In spite of its importance, little empirical studies were conducted to examine how frequently it is used, and especially, in what combinations of circumstances (i.e., beyond the likelihood of being subjected to a clinical override based on a characteristic examined individually) CJPs rely on this form of discretionary power. Therefore, the current study, above all, aims to fill this void regarding the clinical override feature of the LS/CMI by exploring offender characteristics, including LS/CMI items, to identify their potential combinations influencing an offender’s likelihood of being subjected to such a discretion. To do so, the first objective of the study is to establish the override’s prevalence and to provide baseline data with respect to its nature and extent (i.e., the override direction mainly experienced by offenders and the number of overridden risk categories). Secondly, using decision tree algorithms that can detect combination(s) of circumstances, the study has for objective to examine whether the decision to rely on the clinical override feature of the LS/CMI is more likely for certain offenders depending on their characteristics. These objectives are designed to answer the following questions: are there convicted offenders who are more or less likely to be the subject of a clinical override and if so, in what circumstances their risk category is modified?

## Method

### Sample

The sample is composed of all individuals convicted of a crime and imposed a provincial sentence in the province of Quebec between 2008 and 2011. Therefore, all individuals included in this sample were either sentenced to community terms (at least 6 months) or incarceration (less than 2 years). All offenders included in the sample have been assessed using the LS/CMI. Furthermore, the current investigation is limited to the intake (beginning of the sentence) risk assessment made by the CJPs. By focusing exclusively on a specific context, it limited the possibility of confounding factors (e.g., dynamic risk factors changed). The final sample included 15,744 individuals of whom the majority were men (*n* = 14,023; 89.1%) and were serving a community-based sentence (61.7%). Also, the average age was 36.2 years (*SD* = 12.1).

### Procedures

Since the enactment of the *Loi sur le système correctionnel du Québec* in 2007, offender correctional file information, namely the LS/CMI completed form(s) and the clinical notes, is computerized and centralized by Quebec’s Ministry of Public Safety. For the current study, a computer analyst grouped and extracted anonymized data from the computerized files of offenders assessed using the LS/CMI between March 10, 2008 and July 6, 2011. These risk assessment protocols were not carried out as part of a research project but as part of the CJP’s professional duties. That assessment is the focus of the current study.

### Measures

#### The Level of Service and Case Management Inventory (LS/CMI)

The LS/CMI is a generic risk assessment instrument combining a risk and needs assessment tool and a risk management and planning tool (Andrews et al., 2004). The psychometric properties of the LS/CMI have been the subject of several empirical studies. The vast majority supported the reliability of the LS/CMI (Girard & Wormith, 2004; Guay, 2016) and its adequate predictive validity (Guay, 2016; Wormith et al., 2012, 2015). However, looking beyond the predictive value of the total raw score of the actuarial component of the LS/CMI, Giguère and Lussier (2016), using the item response theory, demonstrated that more than half of the LS/CMI items do not significantly discriminate recidivists from non-recidivists. In the context of the current study, these observations are important since (1) individual items of the instrument weighted separately could lead a CJP to use the clinical override to modify an offender’s risk category and (2) CJPs may rely on the LS/CMI to structure their judgment about the offenders’ risk, their treatment/intervention needs, and the adequate modalities of these interventions. Given the importance of this assessment in correctional practices, the LS/CMI was examined in various ways to learn more about the clinical override, that is, the (a) individual items, (b) actuarial score, and (c) cut-point score.

##### LS/CMI items

Although the LS/CMI typically has 11 sections, only items pertaining to the tool’s first six sections were analyzed^3^ with the remaining sections being used for administrative purposes. More precisely, 130 individual items were used for the current study, the majority being found in the tool’s first section—also known as the actuarial evaluation. This section is composed of 43 items divided into 8 subscales of risk and need factors: Criminal History (8 items), Education/Employment (9 items), Family/Marital (4 items), Leisure/Recreation (2 items), Companions (4 items), Alcohol/Drug Problem (8 items), Procriminal Attitude/Orientation (4 items), and Antisocial Pattern (4 items). Twenty items were added to the 43 initial items in section 1 of the tool to detail and clarify the latter. The clarifications provided an idea of the extent and severity of a risk and/or need factor.

While section 1 is the actuarial component of the instrument, the following sections should be seen more as an *aide-mémoire* as it includes other relevant information to establish the offenders’ risk, but especially needs and responsivity. Section 2 “Specific Risk and Need Factors” discusses specific personal problems with criminogenic potential (14 items, such as: trouble conforming, inappropriate sexual activities, racist, or sexist behaviors) in addition to addressing the offender’s history of perpetration for various types of crime (21 items, such as: sexual assault, arson, terrorist activities, DUI). Section 4 “Other Client Issues” refers to other need factors likely to affect the offender’s behavior and risk management in detention and in the community (21 items, such as: finance, immigration, parental situation, mental health, and victimization). Finally, section 5 “Special Responsivity Considerations” includes 11 items which are elements to consider in establishing the offender’s treatment and supervision mode (e.g., denial/minimization, poor motivation, low intelligence). No scores for any of these items are relevant for the actuarial calculation in section 1. In other words, the CJP only notes whether the item is absent or present. Therefore, all items in sections 1 to 5 (except for a few clarifying variables in section 1 which are discrete quantitative) are dichotomously coded (0 = no/absent; 1 = yes/present).

##### Actuarial score

The actuarial score refers to the total score of the LS/CMI’s 43 items in the first section. This total score represents a key aspect of the study given that when a CJP uses the clinical override, it implies that he or she disagrees with the meaning of that score. Hence, it is imperative to investigate its importance by examining whether the probabilities of being subjected to a clinical override are relatively equal across the continuum of LS/CMI total scores. Note that for risk classification purposes, according to the guidelines of the LS/CMI user guide (Andrews et al., 2004), the actuarial score is transferred to a risk category (0–4 = very low; 5–10 = low; 11–19 = moderate; 20–29 = high; 30 and over = very high). According to the LS/CMI user guide, these risk categories should reflect different considerations in terms of risk management strategies. Even if these risk categories are not the subject of particular examinations, they are relevant in the present study in the fact that the authors will sometimes refer to them to express the actuarial score in qualitative terms.

##### Cut-point score

Prior studies (Gore, 2007; Guay & Parent, 2018) have shown that, when the total score is close to the previous or subsequent risk category, CJPs are more inclined to use the clinical override. To examine this possibility, two additional variables were created. The first, “Score separating from the previous risk category,” refers to the difference between the offenders’ actuarial score and the cut-off score of the previous risk category (e.g., an individual with a moderate actuarial score of 11 would be 1 point-score away from the previous risk category labeled “low,” whose scores vary between 5 and 10). The second, “Score separating from the subsequent risk category,” concerns the difference in raw score between the actuarial score and the cut-off score of the subsequent risk category (e.g., the same individual as in the previous example would be 9 point-score away from the subsequent risk category labeled “high,” whose scores vary between 20 and 29).

##### Clinical override

The clinical override corresponds to the sixth section of the LS/CMI. The purpose of this study is to examine the prevalence of this discretion as well as the circumstances affecting an offender’s likelihood to experience one. With that in mind, two dependent variables will be used regarding the clinical override. A variable refers to the presence of a clinical override in the offender’s file (0 = no; 1 = yes). A second variable addresses the direction of the clinical override (0 = downward override; 1 = no override in file; 2 = upward override). Recall that these clinical overrides relate to real-life situations faced by CJPs at the start of the offender’s sentence when the former might have limited knowledge about the offender as well as no, or little, information regarding his/her behavior during the course of the supervision/sentence.

### Analytical Strategy

#### Conceptual model

The conceptual model of the study included a broad range of offender characteristics given that the circumstances surrounding the use of a clinical override could be manifold. In that context, the conceptual model included sociodemographic characteristics (offender’s age at the time of assessment, sex, education level, and work status), current conviction information (nature of the index offense conviction at the time of the LS/CMI assessment and type of sentence),^4^ risk and needs assessment and management components (juvenile and adult prior convictions as well as the cut-point variables), LS/CMI items and actuarial score of each of the offenders. These factors are interpreted in the current study as potential circumstances influencing the utilization of the clinical override. This conceptual model was examined using a series of decision tree algorithms (DTA).

#### Decision tree algorithms

To investigate the covariates of the clinical override feature of the LS/CMI, a series of DTA were conducted. DTA represent an ensemble of statistical methods (e.g., Kass, 1980) specifically designed for data mining as well as for classification and prediction purposes (e.g., Lussier et al., 2019). This method is designed in such a way as to allow the detection of combinations of circumstances in which a clinical override occurred.

##### Rationale

There are several distinctive features about DTA that made it the method of choice for this current study as opposed to other methods (e.g., logistic regression, discriminant function analysis). First, DTA are designed in such a way that they can handle a large number of independent variables (i.e., covariates) of various formats (e.g., nominal, ordinal, continuous). This is particularly important given that, when completing their risk assessment, CJPs have to collect, assess, and consider a large body of information. Second, DTA involve an iterative procedure at each branch of the tree starting with the covariate having the strongest association with the predicted outcome. In doing so, the iterative process allows the identification of nodes, conditions, and paths. There are three types of nodes (e.g., Song & Ying, 2015): (a) root nodes represent the initial subdivision of all subjects into two or more subsets, (b) leaf nodes refer to the result of a combination of decisions, events, or circumstances, and (c) all other nodes, also called internal nodes, are those that connect root to leaf nodes. This partitioning process, therefore, allows the detection and identification of interaction effects underlying the data that may be hardly exposed using traditional regression methods. This feature is particularly relevant in the context of the study given that a distinctive combination of circumstances may have led to a clinical override. Finally, DTA allow several variable combinations to classify an individual due to the contingent nature of this classification model (e.g., see Steadman et al., 2000). Indeed, DTA imply that the interactions between the independent and dependent variables do not result from a simple addition, but from a much more complex process depending on the value of the variables involved. This method differs from the often-used regression methods since the latter suggests that each subject is treated using the same set of variables of which each value will be weighted for classification (Steadman et al., 2000).

##### Method

There are several types of DTA with various strengths and limitations (e.g., Kass, 1980). For the current study, the Classification and Regression Tree (CART; Breiman et al., 1984) was used to examine the covariates of the clinical override. CART is a binary recursive partitioning procedure (using the Gini index) that can handle both nominal, ordinal, and continuous variables. The decision tree is grown to a maximal size until there is no additional split possible. The decision tree is then pruned back, node by node, by removing those that contribute less to the overall performance of the model to limit issues of overfitting. In addition to the pruning procedure, the maximum depth of the DTA has been set to three nodes since deep DTA provide precise but extremely rare subgroups, which also limits the generalization of the results. Therefore, the CART method maximizes the identification of homogenous subgroups, which in the context of the study, means that the method aims to identify combinations of offender characteristics discriminating OSCO from individuals not subjected to an override. All statistical analyses were performed using SPSS 25.0.

##### Validation

Each DTA were subjected to a cross-validation procedure. This cross-validation method was conducted using validation subsamples (*k* = 4) of equal size (*n* = 3,936; 25% of the total sample). These subsamples were selected at random and were treated as an independent sample. For each of them, a tree was conceived with the remaining 75% of cases. Thus, each subsample served as a validation for the 75% that made up the DTA. This procedure was repeated until each subgroup acted as a test group once. These four trees were then combined to produce a final validated tree which was reported and analyzed.

#### ROC curves

Analyses were conducted to assess the predictive validity of the offender characteristic model for the dichotomous dependent variable, that is the presence of a clinical override in the offender’s file. ROC curves (AUC) plot the sensitivity as a function of specificity (Hanley & McNeil, 1982). The AUC varies between 0 and 1 where 0.5 corresponds to chance level. AUCs were interpreted using Rice and Harris’ (2005) criteria (0.56, 0.64, and 0.71 are respectively considered low, moderate, and high).

## Results

### Prevalence

The clinical override appeared to be a very little used discretion. In fact, only 4.1% of assessed offenders (*n* = 650) were subjected to an override over the 3-year data collection stage. More precisely, during this period, the proportion of clinical overrides within the Quebec correctional services varied between 3.6% and 4.8%. Its use peaked in 2008, the year the LS/CMI was implemented by the correctional services and stabilized in the following years. Among OSCO, a majority (*n* = 497; 76.5%) experienced a risk category increase. Hence, less than a quarter (*n* = 153; 23.5%) of clinical overrides were used to decrease the offender’s risk category. Regarding the magnitude of the deviation, most OSCO (*n* = 587; 90.3%) saw their risk category move by the CJP to an adjacent category on the LS/CMI risk category continuum. On the one hand, when the risk category is reviewed in a downward fashion by the CJP, the newly-attributed risk category was almost always changed to the immediate previous category on the tool’s risk continuum (99.3%; *n* = 152). On the other hand, a significant proportion of upwardly overridden cases (12.5%; *n* = 62) were deviated by two risk categories (e.g., a low-risk becomes a high-risk).

### Offender Characteristics Related to the Clinical Override

Bivariate analyses to identify offender characteristics associated with the utilization of a clinical override were conducted. Note that analyses regarding the presence of a clinical override in the file were carried out using the full sample (*n* = 15,744), while those addressing the direction of the override were produced using only OSCO (*n* = 650).^5^ Regarding sociodemographic characteristics, the results of the chi-square tests carried out authorized the rejection of the null hypothesis for all variables except the offender’s sex (Table 1). It appeared that young people (18–25 years old) were rarely subjected to a clinical override. As offenders were older at assessment, this tendency to modify the risk category, especially in an upward fashion, increased slightly and peaked at the oldest offender group. Also, offenders who have their high school diploma were not only more often subjected to a clinical override (6.4%), they tended to be more frequently targeted by an upward override (90.1%) compared to those who do not have their high school diploma (62.1%). A similar trend was observed regarding the work status. The regularly employed offenders being more subjected to a clinical override and an upward override than frequently unemployed individuals.^6^

**Table 1.** Proportion of Clinical Overrides According to Sociodemographic Characteristics.

	Clinical override in file (*n* = 15,744)			Direction of the clinical override (*n* = 650)		
	No	Yes	χ^2^	Upward	Downward	χ^2^
Sex			0.06			4.11
Male	95.9	4.1		77.6	22.4	
Female	96.0	4.0		66.7	33.3	
Age group			72.79***			19.77***
18–25	97.2	2.8		61.5	38.5	
26–40	96.4	3.6		74.9	25.1	
41–55	95.1	4.9		82.0	18.0	
56+	91.7	8.3		83.9	16.1	
Education level			101.87***			70.46***
Completed H.S.	93.6	6.4		90.1	9.9	
Did not complete H.S.	97.0	3.0		62.1	37.9	
Work status			205.42***			74.71***
Regularly employed	93.5	6.5		84.5	15.5	
Frequently unemployed	98.1	1.9		50.6	49.4	

*Note*. H.S. = high school.

*** *p* < .001.

Next, descriptive analyses using two LS/CMI features were performed. First, independent samples *t* tests regarding the 130 LS/CMI items revealed that few significant relationships at the *p* < .001 threshold were found.^7^ Of the 130 items tested, 50% were significant at the *p* < .001 level. A summary of these analyses and the distribution of the effect sizes (Cohen’s *d*; Cohen, 1988)^8^ are presented in Table 2. Among the 130 items examined, most were showing an absence or a small group difference between those subjected to a clinical override and those who were not subjected to one. The most significant differences between OSCO and individuals not subjected to a clinical override were found within section 1 of the LS/CMI, which refers to the actuarial component of the instrument (e.g., being frequently unemployed, *d* = 0.66; currently having a drug problem, *d* = 0.68). More precisely, these findings showed that CJPs tended to rely on the clinical override feature of the LS/CMI with offenders who do not exhibit empirically validated criminal recidivism risk factors.

**Table 2.** Summary of Exploratory Analyzes Between LS/CMI Items and the Use of the Clinical Override.

LS/CMI section—items	LS/CMI items analyzed (*n*)	Distribution of effect sizes according to Cohen’s *d* coefficients		
		Weak	Moderate	Large
Clinical override in file (*n* = 15,744)				
Section 1: Actuarial Tool	63	38 (60.3)	18 (28.6)	0 (0.0)
Section 2.1: Personal problems with criminogenic potential	14	2 (14.3)	0 (0.0)	0 (0.0)
Section 2.2: History of perpetration	21	4 (19.0)	0 (0.0)	0 (0.0)
Section 4: Other client issues	21	3 (14.3)	0 (0.0)	0 (0.0)
Section 5: Special responsivity considerations	11	0 (0.0)	0 (0.0)	0 (0.0)
Total	130	47 (36.2)	18 (13.8)	0 (0.0)
Direction of the clinical override (*n* = 650)				
Section 1: Actuarial tool	63	13 (20.6)	19 (30.2)	8 (12.7)
Section 2.1: Personal problems with criminogenic potential	14	1 (7.1)	4 (28.6)	0 (0.0)
Section 2.2: History of perpetration	21	1 (4.8)	0 (0.0)	0 (0.0)
Section 4: Other client issues	21	0 (0.0)	0 (0.0)	0 (0.0)
Section 5: Special responsivity considerations	11	0 (0.0)	0 (0.0)	1 (9.1)
Total	130	15 (11.5)	23 (17.7)	9 (6.9)

*Note.* The values in the cells indicate *n* (% of row). Cohen’s *d*: Weak = 0.20–0.49; Moderate = 0.50–0.79; Large = 0.80 and over.

Since it was possible that within-group differences between those subjected to an upward and a downward override confounded the findings above, the same analyses were conducted but comparing these two groups subjected to a clinical override (Table 2). The majority of statistically significant differences were still observable within the first section of the LS/CMI. However, large effect sizes were now apparent, but again, this included items found in the actuarial component of the instrument (e.g., being currently unemployed, *d* = 1.10; history of drug problems, *d* = 1.22). Exceptionally, an item within section 5, addressing the offender’s responsivity to treatment, generated a large effect size as well (i.e., denial/minimization, *d* = 0.83). These findings suggested that offenders who were subjected to a downward clinical override, compared to those subjected to an upward override, were more likely to be characterized by risk factors as portrayed by the LS/CMI (using the previous examples, individuals subjected to a downward override were more likely to be unemployed, have a history of drug problems, and deny or minimize their crime).

Second, *t* tests were also conducted using the offender’s LS/CMI actuarial score. The results supported the previous ones regarding the LS/CMI items in that the actuarial score, and therefore, the first section of the LS/CMI was significantly related to the decision to rely on the clinical override feature of the instrument. In fact, the actuarial score had the largest group difference in terms of effect size among all investigated variables. It appeared that OSCO had lower actuarial scores than those not clinically overridden [*t* (733.51) = 25.97, *p* < .001, *d* = 1.04, *n* = 15,744]. More specifically, within OSCO (*n* = 650), offenders whose risk category was revised downward had a higher actuarial score than those whose risk category had been increased [*t* (231.80) = 14.03, *p* < .001, *d* = 1.30].^9^

As for the current conviction information, results are presented in Table 3. Especially, concerning the offender’s type of sentence, probationers were more likely to be subjected to a clinical override than their incarcerated counterparts [χ^2^ (1) = 85.39, *p* < .001, *d* = 0.15, *n* = 15,744]. However, no such statistically significant relationship was reached with regards to the override’s direction. Subsequently, the results indicated that individuals convicted of a sexual [χ^2^ (1) = 308.97, *p* < .001, *d* = 0.28, *n* = 15,744] or a violent [χ^2^ (1) = 18.58, *p* < .001, *d* = 0.07, *n* = 15,744] crime at the time of the LS/CMI assessment were more likely to be subjected to a clinical override than individuals who have been convicted for a non-violent non-sexual crime. Individuals convicted for a violent or sex offense were also more likely to have been subjected to an upward override, while other offenders were more likely to experience a downward override. The reliance on the override feature of the LS/CMI also varied according to risk and need assessment and management components. It appeared that OSCO had less prior juvenile [*t* (780.73) = 6.61, *p* < .001, *n* = 15,744] and adult convictions [*t* (763.54) = 10.49, *p* < .001, *n* = 15,744]. Among OSCO only (*n* = 650), individuals with fewer prior contacts with the criminal justice system were more likely to see their risk category *increase*. Regarding the cut-point variables, some statistically significant differences were observed. Particularly, the closer the offender’s actuarial score approached the previous risk category, the more likely that person was to be subjected to a clinical override, especially in a downward fashion. In addition, when analyzing OSCO only, offenders who have been subjected to a risk category increase were closer to the threshold of the subsequent risk category [*t* (194.39) = 11.04, *p* < .001, *n* = 650] than downwardly overridden offenders.

**Table 3.** Clinical Overrides’ Relation With Characteristics Regarding the Offender’s Latest Conviction and Risk Assessment and Management.

	Total (*n* = 15,744)	Clinical override in file			Direction of the clinical override		
		No (*n* = 15,094)	Yes (*n* = 650)		Downward (*n* = 153)	Upward (*n* = 497)	
	*M* (*SD*)/%	*M* (*SD*)/%	*M* (*SD*)/%	*d*	*M* (*SD*)/%	*M* (*SD*)/%	*d*
*Current conviction information*							
Type of sentence (probation)	61.7	60.9	78.9	.15***	84.3	77.3	.15
Nature of the index offense conviction							
Sexual crime	3.3	2.8	15.4	.28***	2.0	19.5	.42***
Violent crime	29.7	29.3	37.2	.07***	25.5	40.8	.27***
Property crime	23.3	23.6	16.6	.07***	27.5	13.3	.33***
Drug-related crime	19.4	19.8	9.7	.10***	22.9	5.6	.51***
Other crime	24.3	24.5	21.1	.03*	22.2	20.7	.03
*Risk assessment and management considerations*							
Number of juvenile prior convictions	0.6 (2.0)	0.6 (2.0)	0.2 (1.3)	.27***	0.6 (1.7)	0.1 (1.2)	.33***
Number of adult prior convictions	6.4 (8.8)	6.5 (8.8)	3.8 (6.3)	.42***	6.1 (7.0)	3.1 (5.9)	.45***
Actuarial score	20.3 (9.3)	20.6 (9.2)	12.7 (7.6)	1.04***	19.6 (7.1)	10.5 (6.4)	1.30***
Score separating from:							
The subsequent risk category	3.7 (3.0)	3.7 (3.0)	3.9 (2.6)	.06	6.1 (3.1)	3.2 (2.0)	1.02***
The previous risk category	4.9 (2.8)	4.9 (2.8)	3.9 (2.7)	.37***	3.4 (2.2)	4.1 (2.8)	.28***

*Note. d* = Cohen’s *d.*

* p < .05. ***p < .001.

### Decision Tree Analyses of the Clinical Override

**Figure 1.**
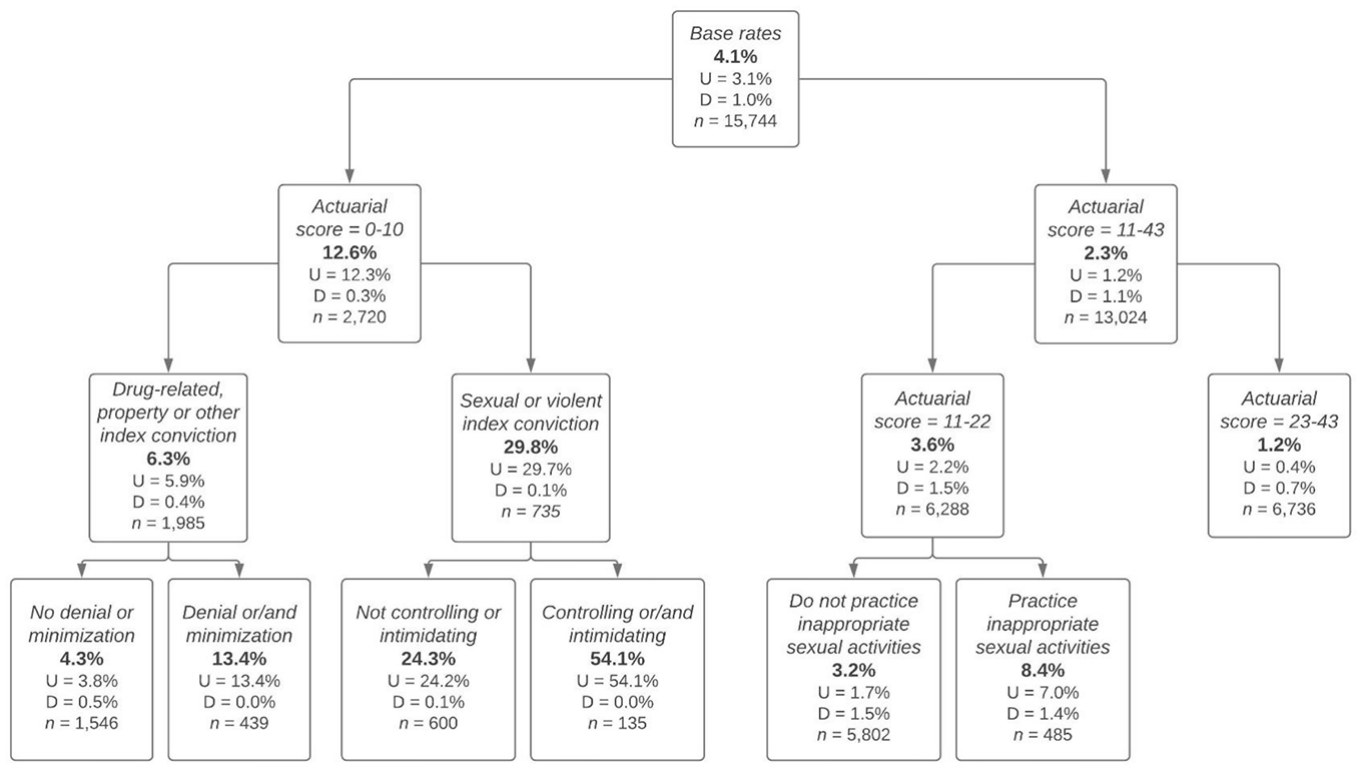
Decision tree of the clinical override according to the offender characteristics. *Note.* The percentages (%) refer to the proportion of OSCO. U = upward override; D = downward override; *n* = number of offenders within the node.

In order to identify the offender characteristics and their possible combinations that were associated with significantly higher/lower likelihood of being subjected to a clinical override, decision tree analyses were carried out. Note that regardless of the dependent variable used (i.e., clinical override in file; direction of the override), the resulting trees were identical. Therefore, only one tree is presented (Figure 1). Using DTA, among the offender characteristics examined, only five were found to influence an offender’s likelihood of being subjected to a clinical override.

Examining those items^10^ revealed that the actuarial score of the LS/CMI was the single most important factor related to the utilization of the clinical override feature of the LS/CMI. The partitioning within the actuarial score occurred between the score 10 and 11, which perfectly divided very low and low-risk offenders (actuarial score = 0–10) from offenders whose actuarial score was associated to the moderate, high, or very high-risk category (actuarial score = 11–43). This division showed that 12.6% of lower risk offenders were subjected to a clinical override compared to 2.3% of the higher risk offenders. Note that further inspection of the decision tree showed that the lower risk group was comprised almost exclusively of individuals whose actuarial risk score was increased by the CJP.

Beyond the actuarial score, there were two second-level factors identified by the DTA. First, in case of lower risk offenders, the second most statistically significant factor that contributed to identify the offender’s likelihood of being subjected to a clinical override was the nature of the index offense. Indeed, 29.8% of lower risk offenders whose index offense conviction was a violent or a sexual crime were subjected to a clinical override, compared to 6.3% for other lower risk offenders. These results showed that only a minority of lower risk offenders were subjected to a clinical override, but among the group of violent and sexual offenders, the rate was significantly higher, seven times higher than the base rate of the full sample. Second, in case of higher risk offenders, the second most statistically significant factor that contributed to the reliance on the override was, again, the LS/CMI actuarial score. The partitioning showed that offenders whose actuarial risk represented a moderate-risk according to the risk continuum (actuarial score = 11–22^11^) were three times more likely (3.6%) than the high and very high-risk group (1.2%) to be subjected to a clinical override. While these differences were statistically significant, the base rate of clinical overrides remained lower than that observed for the full sample.

Finally, the decision tree analyses showed third-level factors statistically associated with the reliance on the clinical override feature of the LS/CMI. While the first two levels of factors were based on hard facts (i.e., actuarial score of the LS/CMI, nature of the index offense conviction), the third level included clinical-based factors that were more subjective in nature, and therefore, relied more on CJP’s clinical judgment. For the lower risk offenders, more specifically, violent offenders (including sex offenders), showing signs of an intimidating or controlling personality further increased the odds of being subjected to a clinical override to reach 54.1%. These individuals, who were 13 times more likely than the full sample to see their risk category modified, were also all subjected to an upward override. As for the lower risk non-violent non-sex offenders, the probabilities of being subjected to this discretion differed little from the base rate. However, among them, those seen as denying or minimizing their offense were more likely to be subjected to an override. In this scenario, the large majority were subjected to an upward override. For the moderate-risk offenders, although upward overrides were typically rare, some individuals stood out. Indeed, offenders who also engaged in inappropriate sexual activities^12^ did undergo an upward override approximately twice as often as the full sample. In addition, for their part, higher risk offenders (actuarial score = 23–43) were almost never subjected to a clinical override. There were no third level factors identified for this group suggesting that clinical-based factors did not weight in compared to the lower risk groups.

The predictive value of the decision tree solution was investigated through ROC curve analysis. According to the area under the ROC curve, the prediction model based on offender characteristics showed a statistically significant ability to discriminate offenders who were and were not subjected to a clinical override. The discriminant value of the predictive model was relatively high (AUC = 0.771; 95% CI = 0.764–0.778).

## Discussion

The clinical override represents an important feature of risk assessment instruments allowing CJPs to use their clinical judgment to revise an adult offender’s risk category. This practice, however, has not been the focus of much empirical research and the current study aimed to fill this gap by examining: (a) its prevalence in a quasi-population of convicted adult offenders assessed with a valid risk assessment tool which includes this feature (i.e., LS/CMI) and (b) a wide range of offender characteristics, including LS/CMI items, and its possible combinations that could influence the likelihood of a given individual to be subjected to such a discretion. In light of the results, it appears that the clinical override is a greatly under-used practice in terms of proportion by CJPs (4.1%). Although relatively rare, it appears that this discretion is used under specific circumstances, and under these, reliance on the clinical override feature of a risk assessment instrument becomes very probable. Indeed, some offenders are more likely than others to be subjected to a clinical override. Especially, it appears that a combination of the actuarial score and the nature of the index offense conviction (without underestimating the over-representation of probationers among OSCO) authorize the identification of offenders more (or less) likely to be subjected to a clinical override (and of the direction of this override). The aforementioned results will be further explored in the paragraphs below in light of previous studies.

### The Clinical Override: A Frequent Phenomenon?

In the current study, the clinical override was a rare phenomenon, perhaps lower than previously observed for a general population of adult offenders (e.g., Brews, 2009; Orton, 2014). This low prevalence supports the idea that CJPs, especially with offenders sentenced to short-term sanctions, tend to approve, or stick with the results of recent actuarial risk assessment tools (Quirion & D’Addese, 2011). However, it seems that the observed rate also complies with the recommendation of the LS/CMI’s architects that it should be a rare practice (i.e., override approximately 5% of cases as reported by Guay & Parent, 2018). Given the very low but relatively stable rate, it appears that this feature is an exceptional measure that should be used under exceptional circumstances.^13^ It remains unclear, however, what those exceptional circumstances are. One hypothesis is that there are institutional/organizational guidelines that may broadly define what those exceptional circumstances are. For example, elsewhere, some have declared that certain offender subgroups (e.g., sex offenders and domestic violent offenders) are mandatory candidates to a clinical override (Carns & Martin, 2011; Cohen et al., 2016; Cole & Angus, 2003). Despite these findings, there is nothing in the research and in the previous scientific literature that suggests that such policies or guidelines exist implicitly or explicitly with respect to this discretion. Furthermore, the current study findings highlight that the clinical override was not used systematically (i.e., in all cases at all times) with a particular offender subgroup.

#### The over-representation of upward clinical overrides

A closer look at the clinical override’s direction further supports the idea that this discretion occurs under relatively specific circumstances. Indeed, the clinical override feature of the LS/CMI was used in general to increase an offender’s risk category beyond what was suggested by the actuarial score of the instrument. This is somewhat of a departure from the scientific literature given that there is no consensus, even if a majority of empirical studies have reported a larger proportion of upward overrides (e.g., Orton et al., 2021; Storey et al., 2012). The preponderance of upward clinical overrides, with respect to the LS/CMI, may be explained by the tool’s user guide which suggests that a downward override cannot exceed a single risk category while an upward override is limitless. This instruction implicitly implies that the actuarial component of the tool might have more issues with false negatives (i.e., offenders whose risk was underestimated) than false positives (i.e., offenders whose risk was overestimated) to the point where it may be necessary to make adjustments based on clinical judgment. This may mislead CJPs in thinking that the use of the upward override is more relevant, and by extension, should be more frequent. Although plausible, this hypothesis does not consider several parameters that could influence the utilization of a clinical override. In fact, an alternative hypothesis is that the clinical override is used, not so much to adjust a risk prediction, but rather as *a risk management intervention strategy* (see also Gore, 2007). An inspection of the combinations of offender characteristics related to the clinical override feature of the LS/CMI provides supporting evidence.

### Clinical Override as a Risk Management Strategy

The study findings suggest that the clinical override is a risk management strategy used in certain circumstances. While risk prediction refers to quantification and qualification of recidivism risk probabilities, risk management refers to criminal justice initiatives, safeguards, and interventions to prevent criminal recidivism (see Heilbrun, 1997). It is argued that the clinical override, therefore, is not so much a reflection that the risk of recidivism is underestimated by the actuarial tool, but rather that the label might not be well-suited for a certain offender according to the CJP’s assessment of the individual. In this regard, Cohen et al. (2016) noted that offenders who had been subjected to an upward override had more contacts with their probation officers as well as more frequent and intense treatment than individuals belonging to their initial risk category. In this context, the clinical override may then be seen as a safety net allowing CJPs to change the offender’s risk category, and by extension, to modulate the intervention to fit this risk. This, in order to restore the discrepancy between risk prediction and risk management in an instrument focused on general recidivism. In other words, risk management-oriented actions may be warranted by CJPs in cases where the risk of reoffending is more specific in nature than what is suggested by the generic risk assessment instrument (e.g., sexual or domestic violence). The seriousness of the threat anticipated—hidden behind the label of general reoffending—overshadows probabilities of reoffending and then justifies more restrictive measures in terms of risk management for certain offenders, particularly with regards to community surveillance.

Several observations seem to support this assertion. Indeed, the use of the clinical override was significantly associated with various offender characteristics (e.g., actuarial score). For example, offenders serving their sentence in the community were more likely to be overridden in an upward fashion since they represented an immediate threat of criminal reoffense compared to those incarcerated (see also, Brews, 2009; Girard, 1999). Furthermore, individuals who had been convicted of a sex crime—and to a lesser extent a violent crime—were more likely to be subjected to an upward clinical override. This tendency to use this discretion specifically with individuals convicted of a sex crime had been reported for adult (e.g., Orton, 2014; Storey et al., 2012) and adolescent offenders (e.g., Carns & Martin, 2011). However, taken individually, these factors do not tell the whole story as the prevalence of the clinical override was very low, much lower than any of these circumstances more typically encountered by CJPs.

Therefore, the decision tree analyses were useful to highlight that it was not a particular factor that led CJPs to rely on the clinical override feature of the LS/CMI, but a combination of a few specific offender characteristics with varying occurrences. These analyses, in particular, identified an offender subgroup whose probabilities of being subjected to a clinical override were not only significantly higher compared to other convicted offenders, but also always used to increase the risk category assigned to these individuals. Based on the study findings, it appeared that low-risk individuals currently serving a sentence for a violent or sex crime were the most likely candidates for a clinical override. Given that a large majority were sentenced to community terms, it seemed that CJPs may be using the clinical override to increase the community-based supervision of these individuals beyond what the actuarial risk assessment suggests. In fact, among the lower risk individuals convicted for a violent or sex crime, it was those showing a negative attitude (i.e., intimidating and controlling) who were most likely to see their risk category increased by CJPs. Such attitudes may be interpreted as red flags about the offenders’ compliance with community-based supervision or treatment. Based on this combination of offender characteristics, the use of the clinical override surpassed the 50% threshold, which was more than 10 times the prevalence observed for the full sample. As others have raised, such combinations of circumstances support the fact that CJPs are not immune to dread factors (e.g., Gigerenzer, 2004). This phenomenon is described as a tendency to put emphasis on frightening, but scarce events (e.g., sexual recidivism of a sex offender) rather than common events characterized by higher occurrence probabilities (e.g., non-recidivism of a sex offender; Hanson & Bussière, 1998; McCann & Lussier, 2008). Therefore, although it seems paradoxical to upwardly override low-risk offenders, this demonstrates that this discretion is an apparatus granting CJPs the opportunity to revise an offender’s risk category under specific circumstances exceeding empirical prediction standards.

### Limitations of the Current Study

Despite the relevant observations, it is important to underline the study’s limitations. First, only one assessment was analyzed, that is, the LS/CMI at intake, despite the presence of other potentially relevant assessments (e.g., mental health). This limit was mitigated by the fact that the current sample was comprised of individuals serving short sentences, which necessarily means fewer assessments. Second, the data considered for this study focused on offender characteristics. Thus, it is quite possible that external factors have played a role in CJP’s decision to rely on the clinical override feature of the LS/CMI. Third, the combinations of offender characteristics identified using DTA did not specifically provide information on the intuitive reasoning behind CJP’s decision. Therefore, there is no evidence indicating that CJPs were aware of the presence and the clarity of the current study’s observations. Fourth, although this was a large and representative sample of offenders serving a provincial sentence in the province of Quebec, it is not possible to determine whether the results were generalizable to other jurisdictions. Fifth, the study does not venture deeply into the “why” of the gap between risk prediction and management when CJPs use the LS/CMI. A thorough investigation of the LS/CMI, including this instrument’s assumptions and one-size-fits-all approach, is then warranted to better contextualize this discrepancy. Sixth, the current study did not investigate whether the clinical override turned out to be a “good decision” (e.g., whether OSCO were less likely to reoffend). Such a conceptualization could lead to misinterpretations without a proper assessment of the consequences of the clinical override on risk management decisions. For example, the decision to change in an upward fashion an offender’s risk category may have a significant impact on preventing criminal recidivism by increasing supervision and treatment intensity. If the strategies proved to be effective, it may lead to empirical observations that those OSCO had lower recidivism rates. These findings may be interpreted erroneously as poor clinical judgment by the CJPs given the low recidivism rates. Hence, researchers going down this path, on whether the clinical override is a warranted discretion, need to be cautious and examine the nature and impact of the strategies put in place as a result of the override.

## Conclusion

This study sheds new light on the use of a discretionary power by CJPs responsible for conducting risk assessments of convicted adult offenders. Although the override is rarely utilized by CJPs using the LS/CMI, the current investigation highlights that this discretion seems to occur in some specific circumstances which could be interpreted as the combination of hard facts and clinical judgment regarding the offender. Indeed, it appears that the use of the clinical override stems from a perceived gap between risk prediction and risk management, particularly among low-risk sexual or violent offenders sentenced to community terms. Therefore, the findings highlight the challenges of risk assessment in the context of community corrections and relatively short sentences, especially for some offenders who are seen as threats to society. However, the current observations are limited in the absence of outcome data as to the impact of a risk category modification (e.g., recidivism, community supervision modalities), as well as clear guidelines given to CJPs regarding the use of the clinical override. In fact, if there are any existing guidelines, their application to real-life situations remains unclear in light of the results presented in this study. Although the clinical override seems more likely in certain combinations of circumstances, there appears to be fluctuations in its use in many cases. Therefore, in the presence of such a black box, it would be desirable to conduct exploratory studies examining the use of the clinical override feature of actuarial instruments from the CJPs’ perspectives. More precisely, it would be interesting to examine the rationale to override and to carry out such a study using a conceptual framework related to experts’ decision-making, biases, and sources of errors (e.g., Dror et al., 2006). After all, although the clinical override is a rare phenomenon, several studies, including this one, cannot rule out the possibility that personal biases and errors might have influenced the CJP’s decision to revise an offender’s risk category. Indeed, it seems that certain offenders are more likely to be subjected to a clinical override. Why is that possible?

## Notes

1. The current issue does not examine whether the clinical override increases or decreases the predictive accuracy of official recidivism in a risk assessment’s instrument.

2. Conversely, Gore (2007) observed that the majority of convicted sex offenders were subjected to a downward clinical override. This is potentially due to the fact that this is the only study where the risk assessment tool was designed specifically to assess sexual recidivism risk among sex offenders.

3. Section 3 “Prison Experience—Institutional Factors” was excluded from the study given that it was only coded for those previously incarcerated.

4. Offender’s age, sex, index offense, and type of sentence are external to the LS/CMI assessment. However, as for the LS/CMI, they are systematically compiled information as the offender enters the criminal justice system.

5. The results addressing the override’s direction should be taken with some hindsight since statistics were calculated based on a reduced sample, in this case only OSCO.

6. To complement these chi-squared tests, standardized residuals were used to determine which cells significantly contributed to the differences between expected and observed values (see Everitt & Skrondal, 2002). Note that OSCO were largely responsible for the statistically significant findings observed, with standardized residuals varying between 1.9 and 10.0 across cells and analyses.

7. Due to the large sample size and the high number of comparisons, only results with a significance level of *p* < .001 were considered statistically significant.

8. The effect sizes of the tests were measured and interpreted using Cohen’s *d* (0.20 = weak; 0.50 = moderate; 0.80 = large; Cohen, 1988).

9. Further inspection of the data also showed that the prevalence of the clinical override significantly varied according to the offender’s initial risk category established by the actuarial section of the LS/CMI [χ^2^ (4) = 651.09, *p* < .001, *n* = 15,744].

10. Analyses were also conducted using the LS/CMI subscales (recognized by the architects of the tool; Andrews et al., 2004) instead of the individual items. The score for each subscale from sections 1 to 5 was obtained through a simple addition to the dichotomous items that compose it (0 = no/absent; 1 = yes/present). The LS/CMI subscales provided excessively similar results and trends to the individual items. While there are some small differences, these lied in the nature of the covariates. Individual LS/CMI items identified as covariates were substituted by the subscale containing them. For instance, the “inappropriate sexual behavior” item was substituted by the subscale “personal problems with criminogenic potential.” These results reiterated the contribution of these covariates, as well as the stability of the observations.

11. Technically, this score range includes few offenders representing a high-risk according to the LS/CMI’s risk category continuum. To avoid unnecessary complications, these lower end high-risk offenders were labeled as moderate.

12. A large proportion (39.3%) being offenders whose index offense was sexual in nature.

13. Given that the study was conducted in one jurisdiction (i.e., Quebec), it was not possible to study allegiance to the LS/CMI. However, it is possible that findings will be different depending on the jurisdiction’s allegiance to the instrument. In fact, this points to the need to conduct more comparative research on the use of the clinical override using the same instrument across jurisdictions.
